# Surgical treatment of isolated cartilage lesions during anterior cruciate ligament reconstruction was associated with greater lesion stability but similar 2‐year clinical outcomes: A registry‐based matched‐pair second‐look study

**DOI:** 10.1002/jeo2.70817

**Published:** 2026-06-30

**Authors:** Iacopo Romandini, Piero Agostinone, Pierre Rotzius, Alexander Sandon

**Affiliations:** ^1^ Centro di ricerca Codivilla‐Putti IRCCS Istituto Ortopedico Rizzoli Bologna Italy; ^2^ Department of Biomedical and Neuromotor Sciences University of Bologna Bologna Italy; ^3^ Sodersjukhuset/Karolinska Institute SÖS Stockholm Sweden; ^4^ Department of Molecular Medicine and Surgery, Stockholm Sports Trauma Research Center Karolinska Institute Stockholm Sweden

**Keywords:** anterior cruciate ligament reconstruction (ACLR), cartilage treatment, knee, Swedish Knee Ligament Registry (SKLR)

## Abstract

**Purpose:**

The management of cartilage lesions encountered during anterior cruciate ligament reconstruction (ACLR) remains debated, with limited evidence on whether surgical treatment affects lesion progression or outcomes. This study aimed to compare structural progression and clinical outcomes of isolated cartilage lesions treated or left untreated during primary ACLR, using data from the Swedish Knee Ligament Registry (SKLR).

**Methods:**

Patients who underwent both primary and revision ACLR between 2005 and 2024 were identified in the SKLR. Inclusion required an isolated cartilage lesion documented arthroscopically at primary ACLR. Multi‐compartment lesions and multi‐ligament procedures were excluded. A 1:1 matched analysis compared patients who underwent surgical cartilage treatment (debridement or microfracture) with untreated patients. Structural evolution was assessed by comparing arthroscopic findings at primary and revision ACLR and classified as stable, progressed or regressed based on International Cartilage Repair Society (ICRS) grade and lesion size. Secondary analyses included 2‐year revision‐free survival and patient‐reported outcomes (Knee injury and Osteoarthritis Outcome Score, EuroQol 5‐Dimension questionnaire, EuroQol Visual Analogue Scale) in patients without revision within 2 years.

**Results:**

A matched cohort of 156 patients (78 treated and 78 untreated) was included. Baseline characteristics were comparable, except for operated side (*p* = 0.007). At revision ACLR, treated patients showed higher lesion stability than untreated patients (21.8% vs. 5.1%; *p* = 0.005). This effect was significant in low‐grade lesions (ICRS I–II), with higher stability (27.1% vs. 5.1%; *p* = 0.002) and lower progression (15.3% vs. 32.2%; *p* = 0.050), while no differences were observed in high‐grade lesions. Two‐year revision‐free survival was comparable (log‐rank *p* = 0.53). Among patients without revision (*n* = 88), both groups improved in patient‐reported outcome measures with no between‐group differences.

**Conclusions:**

Surgical treatment of isolated cartilage lesions during primary ACLR was associated with greater structural stability at revision surgery, particularly in low‐grade lesions, without superior short‐term clinical outcomes. These findings suggest structural benefits may not translate into early improvement.

**Level of Evidence:**

Level II, prospective comparative study.

## Graphical abstract

Graphical abstract summarizing the study design and main findings. Patients undergoing primary and revision ACL reconstruction in the Swedish Knee Ligament Registry were screened to identify isolated cartilage lesions, followed by 1:1 matching between treated and non‐treated patients. Surgical treatment of isolated cartilage lesions was associated with greater structural stability at revision ACLR, particularly in low‐grade lesions, whereas revision‐free survival and 2‐year patient‐reported outcomes were comparable between groups.

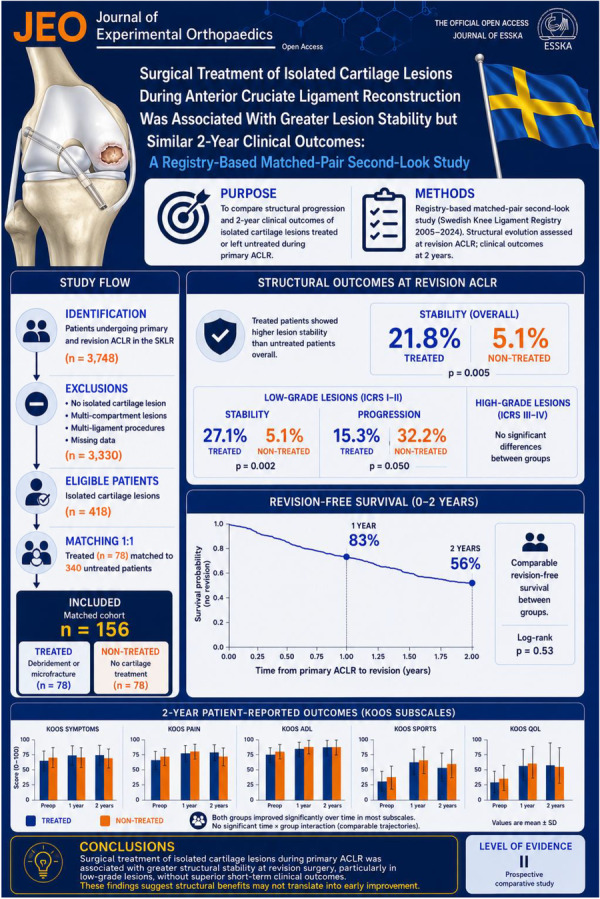

**Abbreviations**
ACL: anterior cruciate ligament
ACLR: anterior cruciate ligament reconstruction
ADL: activities of daily living
BTB: bone–patellar tendon–bone
CI: confidence interval
EQ‐5D: EuroQol 5‐Dimension questionnaire
EQ‐VAS: EuroQol Visual Analogue Scale
HT: hamstring tendon autograft
ICRS: International Cartilage Repair Society
KOOS: Knee injury and Osteoarthritis Outcome Score
LFC: lateral femoral condyle
LP: lateral patella
LTP: lateral tibial plateau
MCL: medial collateral ligament
MFC: medial femoral condyle
MTP: medial tibial plateau
NA: not applicable
OA: osteoarthritis
OCD: osteochondritis dissecans
OR: odds ratio
PCL: posterior cruciate ligament
PLC: posterolateral corner
PROMs: patient‐reported outcome measures
PTOA: post‐traumatic osteoarthritis
QoL: quality of life
RTS: return to sport
SKLR: Swedish Knee Ligament Registry

## INTRODUCTION

Anterior cruciate ligament (ACL) injuries are among the most common orthopaedic conditions in young, physically active individuals [26]. These injuries are often accompanied by intra‐articular damage, particularly cartilage lesions, which may result from acute traumatic impact or develop progressively due to chronic joint instability [3]. Although early magnetic resonance imaging (MRI) may not always visualize such defects, bone marrow oedema, especially in the lateral compartment, can serve as an indirect marker of chondral trauma and has been associated with subsequent cartilage degeneration [6, 24]. This is not a minor clinical concern. Epidemiological studies estimate that between 16% and 46% of patients undergoing ACL reconstruction (ACLR) present with concomitant cartilage injury [19, 30]. Lesions involving the medial femoral condyle (MFC) are particularly troublesome, being linked to impaired joint function, poorer patient‐reported outcome measures (PROMs), increased risk of post‐traumatic osteoarthritis (PTOA) and lower rates of return to sport (RTS) [9, 16, 32, 37].

The management of cartilage lesions encountered during ACLR remains controversial, with limited high‐quality evidence guiding intraoperative decisions. Therapeutic options range from simple debridement to marrow‐stimulating techniques such as microfracture, and in selected cases, restorative options including autologous chondrocyte implantation (ACI) or osteochondral autograft transplantation (OAT) [20]. While some studies suggest that treating cartilage lesions at the time of ACLR may improve outcomes, others report comparable or even inferior results compared to leaving the lesions untreated [3, 7, 13]. A recent meta‐analysis confirmed the negative impact of concomitant cartilage damage on clinical outcomes after ACLR but failed to demonstrate a clear benefit of surgical intervention compared with observation [15]. These concerns are particularly relevant in young, active individuals, who are more vulnerable to early‐onset PTOA [4, 17]. Given this persistent ambiguity, there is a clear need for large‐scale, comparative studies evaluating whether surgical treatment of concurrent cartilage defects at the time of ACLR influences long‐term structural and functional outcomes.

The present registry‐based matched‐pair study therefore aimed to compare the structural progression and clinical outcomes of isolated cartilage lesions that were either surgically treated or left untreated during primary ACLR, using matched data from the Swedish Knee Ligament Registry (SKLR). Cartilage status was evaluated arthroscopically at both primary and revision ACLR, and PROMs were analysed longitudinally for up to 2 years. It was hypothesized that surgical treatment of isolated cartilage lesions at the time of primary ACLR would be associated with lower rates of lesion progression and superior PROMs compared to non‐treatment.

## METHODS

### Study design and data source

The cohort study used prospectively collected data from the SKLR. Established in 2005, the registry captures more than 90% of all ACLR performed nationwide annually [34]. Its primary purpose is to improve the management of ACL injuries by providing hospitals and clinics with systematic feedback on surgical techniques and outcomes, and by identifying prognostic factors for both favourable and poor results [34]. The registry integrates two complementary data sources. The primary source is completed by healthcare professionals and includes patient demographics, injury‐related factors and treatment‐specific details for both surgical and non‐surgical management of ACL injuries. The secondary source consists of patient‐reported data, collected through validated outcome instruments: the Knee injury and Osteoarthritis Outcome Score (KOOS), which evaluates five subscales of knee health [29]; the EuroQol 5‐Dimension questionnaire (EQ‐5D) and the EuroQol Visual Analogue Scale (EQ‐VAS), which assess health‐related quality of life (QoL) [1] and a general health status rating.

### Ethics

The analysis of the SKLR is approved by the Regional Ethics Committee in Stockholm (ID 2011/337‐31/3). The registry complies with Swedish data security legislation. Participation in the SKLR is voluntary for both patients and surgeons, and no written consent is required for the use of national registry data in Sweden. The study was also approved by the SKLR steering committee.

### Patient selection and matching

All patients in the SKLR who underwent both primary and revision ACLR between 1 January 2005, and 31 December 2024, were screened for eligibility. Patients were included if an isolated cartilage lesion was documented arthroscopically at the time of primary ACLR and if pre‐operative PROMs were available. Patients were excluded if cartilage lesions involved more than one compartment, if the primary ACLR included multi‐ligament reconstruction involving the posterior cruciate ligament (PCL), posterolateral corner (PLC) or medial collateral ligament (MCL), or if key information on cartilage lesion status, mechanism of injury, PROMs, ACL graft type or injury date was missing.

A 1:1 matching was then performed to generate two comparable groups: patients who underwent surgical treatment of cartilage lesions at the time of primary ACLR (treated group) and those whose lesions were left untreated (non‐treated group). Exact matching was applied for sex, presence of meniscal lesion, meniscectomy, meniscal suture and cartilage lesion grade using the International Cartilage Repair Society (ICRS) grading system [2]. Caliper matching was used for age (± 5 years) and time from injury to primary ACLR (± 12 months, extended to ±24 months if no suitable candidate was found). When multiple potential matches were available, the nearest‐neighbour method was used on continuous variables, including time from index injury to revision ACLR, as a tiebreaker. No replacement was permitted.

### Outcomes

Structural outcomes were assessed intraoperatively by comparing cartilage lesion characteristics recorded at primary ACLR with those recorded at revision ACLR in patients who underwent both procedures. This approach was chosen because arthroscopic evaluation remains the clinical reference standard for cartilage assessment [33]. Cartilage lesions were classified according to anatomical location (medial and lateral femoral condyle – MFC/LFC, medial and lateral tibial plateau – MTP/LTP, trochlea, medial and lateral patella – MP/LP), size (<2 or ≥2 cm^2^) and severity using the ICRS grading system (Grades 1–4) [2] recorded in the registry. Lesion evolution between primary and revision ACLR was categorized as:

‐ Stable: both ICRS grade and lesion size category remained unchanged;
‐ Progressed: increase in ICRS grade and/or in lesion size category;
‐ Regressed: decrease in ICRS grade and/or lesion size category.

If one parameter improved and the other worsened, the lesion was classified as progressed. This definition reflects a change in arthroscopic grading rather than histological restoration of native cartilage and should be interpreted as a morphological rather than biological modification.

The primary outcomes of the study were:

‐ Comparative analysis of cartilage lesion characteristics (site, grade, size and type of surgical treatment such as debridement or microfracture) between treated and non‐treated groups at the time of primary ACLR.
‐ Evaluation of the structural evolution of cartilage lesions between primary and revision ACLR, categorized as stable, progressed or regressed based on changes in ICRS grade and lesion size.
  Secondary outcomes included:
‐ Time to revision ACLR, analysed using Kaplan–Meier survival estimates, with particular attention to failures occurring within the first 2 years after primary surgery.
‐ Clinical outcomes following primary ACLR were assessed using the KOOS (all five subscales), the EQ‐5D, the EQ‐VAS and a self‐reported general health status rating. PROMs were analysed both as absolute values and as changes from baseline (delta values). The analysis included only patients who completed the 2‐year follow‐up with available and complete PROMs data. In line with the methodology described by Rizvanovic et al. [28], the study cohort for PROMs analyses consisted of all patients with available 2‐year clinical data after primary ACLR who had not undergone revision surgery.

### Statistical analysis

The sample size was determined by performing a power analysis on the change in the KOOS Pain score from preoperative to 2‐year follow‐up. A previous study revealed a standard deviation of 15 points for the KOOS Pain subscale in patients undergoing ACLR with or without concomitant cartilage treatment [30]. With an alpha of 0.05 and a power of 0.80, and a clinically significant difference of 10 points for the KOOS Pain, the minimum sample size required was 36 patients per group, for a total of 72 patients. Considering a 15% potential dropout rate, 43 patients were needed per group, for a total of 86 patients. The quality of the matching procedure was verified by comparing baseline demographic and surgical variables between the treated and non‐treated groups. Differences were assessed to ensure adequate balance across matched pairs. Categorical variables were summarized as frequencies and percentages, and continuous variables as means with standard deviations or medians with interquartile ranges, depending on data distribution. Between‐group comparisons for categorical variables were performed using chi‐square or Fisher's exact tests, as appropriate. Continuous variables were compared with independent‐samples *t* tests or Mann–Whitney *U* tests for non‐normally distributed data. Univariate regression analyses were conducted to compare baseline characteristics of the treated and non‐treated groups and confirm the absence of significant demographic or surgical differences. Comparative analyses were also performed to characterize cartilage lesions (location, grade, size, type of treatment) and their evolution (progression, stability, regression) between groups, with odds ratios (ORs) and 95% confidence intervals (CIs) reported. Clinical outcomes (KOOS subscales, EQ‐5D, EQ‐VAS and health status) were analysed both within and between groups. Within‐group changes over time were assessed using repeated‐measures analysis of variance, which accounts for correlations between repeated measures within the same subject and evaluates the overall temporal trend across all follow‐up points (preoperative, 1 year and 2 years). Time × group interaction effects were evaluated to determine whether outcome trajectories differed between treated and non‐treated patients. When a significant time effect was detected, pairwise post hoc comparisons with Sidak correction were performed. All statistical analyses were performed using IBM SPSS Statistics, Version 25.0 (IBM Corp.). Statistical significance was set at a two‐tailed *p* value < 0.05.

## RESULTS

### Patient characteristics

**Figure 1:**
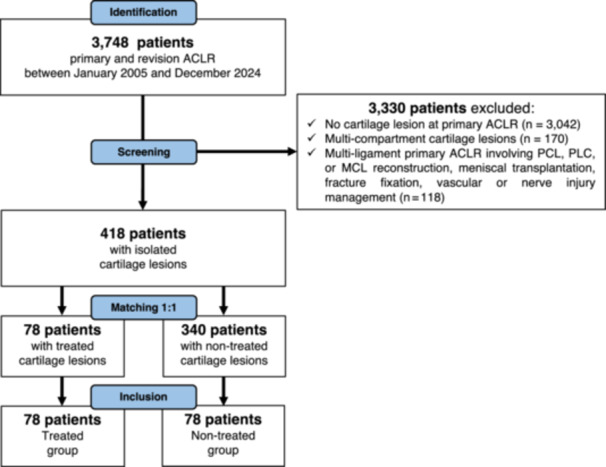
Flowchart of patient selection, exclusions, matching and final inclusion (*n* = 156). ACLR, anterior cruciate ligament reconstruction; MCL, medial collateral ligament; PCL, posterior cruciate ligament; PLC, posterolateral corner.

A total of 3748 patients who underwent both primary and revision ACLR between 2005 and 2024 were identified in the SKLR. After applying the exclusion criteria, 418 patients with isolated cartilage lesions were eligible. Of these, 78 had undergone surgical treatment of the lesion and 340 were left untreated at the time of the primary ACLR. Following a 1:1 matching procedure, 156 patients (78 treated group and 78 non‐treated group) were included in the final analysis (Figure 1). No significant differences were found in the matching variables, except for the operated side (OR = 2.44; 95% CI, 1.28–4.67; *p* = 0.007) (Table 1).

**Table 1:** Baseline demographic and surgical characteristics of the matched cohort.

Characteristic	Treated (*n* = 78)	Non‐treated (*n* = 78)	OR (95% CI)	*p* Value
Sex (male)	52 (66.7%)	52 (66.7%)	1.00 (0.51–1.95)	1.000
Side (left)	44 (56.4%)	27 (34.6%)	2.44 (1.28–4.67)	**0.007**
Age at primary surgery (years)	25.2 ± 7.7	24.1 ± 8.1	1.02 (0.98–1.05)	0.444
Age at revision (years)	28.5 ± 8.7	27.4 ± 9.1	1.01 (0.98–1.05)	0.444
Time injury → primary ACLR (months)	15.4 ± 33.2	12.1 ± 14.9	1.01 (0.99–1.02)	0.430
Time primary → revision ACLR (years)	3.5 ± 3.0	3.4 ± 2.6	1.02 (0.91–1.14)	0.748
Time index injury → revision ACLR (years)	4.9 ± 4.1	4.4 ± 3.2	1.04 (0.95–1.13)	0.405
Meniscal lesion (yes)	53 (67.9%)	47 (60.3%)	1.40 (0.73–2.70)	0.317
Meniscectomy (yes)	40 (51.3%)	39 (50.0%)	0.95 (0.51–1.78)	0.873
Meniscal suture (yes)	9 (11.5%)	9 (11.5%)	1.00 (0.37–2.67)	1.000
Graft type	70 HT (89.7%)	72 HT (92.3%)	NA	0.623
	6 BTB (7.7%)	4 BTB (5.1%)		
	2 Other (2.6%)	2 Other (2.6%)		
Contact sport	55 contact (70.5%)	57 contact (73.1%)	NA	0.819
	18 no contact (23.1%)	15 no contact (19.2%)		
	5 Other (6.4%)	6 Other (7.7%)		

*Note*: Values are expressed as mean ± standard deviation for continuous variables and as number (%) for categorical variables. Values in bold indicate statistically significant differences (*p* < 0.05).

Abbreviations: ACLR, anterior cruciate ligament reconstruction; BTB, bone–patellar tendon–bone autograft; CI, confidence interval; HT, hamstring tendon autograft; NA, not applicable; OR, odds ratio.

### Cartilage lesions

#### Lesions characteristics

At the time of the primary ACLR, no significant differences were observed between groups in terms of lesion site, size, ICRS grade or aetiology (Table 2). The MFC was the most frequently involved location in both cohorts (75.6% in each group), with identical distributions across all compartments. Lesion size (<2 vs. ≥2 cm^2^) did not significantly differ between treated and non‐treated patients (79.5% vs. 66.7% for lesions <2 cm^2^; *p* = 0.073). Likewise, ICRS grade distribution was comparable between groups (*p* = 1.000), as was lesion aetiology (*p* = 0.382). An exploratory univariate regression analysis was performed to assess potential correlations between demographic variables and cartilage lesion characteristics: no significant associations were identified.

**Table 2:** Cartilage lesion characteristics at the time of primary ACLR.

Characteristic	Treated (*n* = 78)	Non‐treated (*n* = 78)	OR (95% CI)	*p* Value
Lesion site				
MFC	59 (75.6%)	59 (75.6%)	1.00 (0.48–2.08)	1.000
LFC	4 (5.1%)	4 (5.1%)	1.00 (0.24–4.15)	1.000
MTP	2 (2.6%)	2 (2.6%)	1.00 (0.14–7.28)	1.000
LTP	5 (6.4%)	5 (6.4%)	1.00 (0.28–3.60)	1.000
Trochlea	2 (2.6%)	2 (2.6%)	1.00 (0.14–7.28)	1.000
Patella	4 (5.1%)	4 (5.1%)	1.00 (0.24–4.15)	1.000
Lesion size				
<2 cm^2^	62 (79.5%)	52 (66.7%)	1.94 (0.94–4.00)	0.073
≥2 cm^2^	16 (20.5%)	26 (33.3%)	0.52 (0.25–1.06)	0.073
ICRS grade				
Grade 1	13 (16.7%)	13 (16.7%)	1.00 (0.42–2.39)	1.000
Grade 2	46 (59.0%)	46 (59.0%)	1.00 (0.36–2.82)	1.000
Grade 3	16 (20.5%)	16 (20.5%)	1.00 (0.17–5.90)	1.000
Grade 4	3 (3.8%)	3 (3.8%)	1.00 (0.17–5.90)	1.000
Aetiology				
Trauma	73 (93.6%)	66 (84.6%)	0.76 (0.42–1.40)	0.382
OCD	2 (2.6%)	9 (11.5%)	0.23 (0.05–1.06)	0.060
OA	1 (1.3%)	2 (2.6%)	0.45 (0.04–5.09)	0.516
Other	2 (2.6%)	1 (1.3%)	1.81 (0.16–20.79)	0.623
Treatment				
None	0	78 (100%)	NA	NA
Debridement	60 (76.9%)	0	NA	NA
Microfracture	18 (23.1%)	0	NA	NA

*Note*: Values are expressed as number (%).

Abbreviations: ACLR, anterior cruciate ligament reconstruction; CI, confidence interval; ICRS, International Cartilage Repair Society; LFC, lateral femoral condyle; LTP, lateral tibial plateau; MFC, medial femoral condyle; MTP, medial tibial plateau; NA, not applicable; OA, osteoarthritis; OCD, osteochondritis dissecans; OR, odds ratio.

#### Lesion evolution

Analysis of lesion evolution between primary and revision ACLR demonstrated a significantly higher rate of stable lesions in the treated group compared to the non‐treated group (21.8% vs. 5.1%; OR 5.16, 95% CI 1.65–16.13; *p* = 0.005). Rates of regression were comparable between groups (61.5% vs. 67.9%; *p *= 0.403), while progression occurred less frequently in the treated cohort (16.7% vs. 26.9%), although this difference did not reach statistical significance (*p* = 0.124) (Table 3a).

**Table 3:** Evolution of cartilage lesions between primary and revision ACLR stratified by ICRS grade.

(a) Overall cohort				
Lesion status	Treated (*n* = 78)	Non‐treated (*n* = 78)	OR (95% CI)	*p* Value
Stable	17 (21.8%)	4 (5.1%)	5.16 (1.65–16.13)	**0.005**
Regressed	48 (61.5%)	53 (67.9%)	0.76 (0.39–1.46)	0.403
Progressed	13 (16.7%)	21 (26.9%)	0.54 (0.25–1.18)	0.124

**(b) ICRS Grade I–II**				
**Lesion status**	**Treated (*n*** = **59)**	**Non‐treated (*n*** = **59)**	**OR (95% CI)**	***p* Value**
Stable	16 (27.1%)	3 (5.1%)	6.95 (1.90–25.37)	**0.002**
Regressed	34 (57.6%)	37 (62.7%)	0.81 (0.39–1.69)	0.403
Progressed	9 (15.3%)	19 (32.2%)	0.38 (0.15–0.93)	0.050

**(c) ICRS Grade III–IV**				
**Lesion status**	**Treated (*n*** = **19)**	**Non‐treated (*n*** = **19)**	**OR (95% CI)**	***p* Value**
Stable	1 (5.3%)	1 (5.3%)	1.00 (0.06–17.25)	1.000
Regressed	14 (73.7%)	16 (84.2%)	0.53 (0.11–2.60)	0.660
Progressed	4 (21.1%)	2 (10.5%)	2.27 (0.36–14.19)	0.660

*Note*: Values are expressed as number (%). Between‐group comparisons were performed using Fisher's exact test, and OR with 95% CI were calculated using univariate logistic regression. Values in bold indicate statistically significant differences (*p* < 0.05).

Abbreviations: ACLR, anterior cruciate ligament reconstruction; CI, confidence interval; ICRS, International Cartilage Repair Society; OR, odds ratio.

When stratified according to lesion severity (Table 3), the structural benefit was confined to low‐grade lesions (ICRS I–II). In this subgroup, treated patients showed a significantly higher rate of lesion stability at revision (27.1% vs. 5.1%; OR 6.95; *p* = 0.002) and a lower rate of progression (15.3% vs. 32.2%; OR 0.38; *p* = 0.050) (Table 3b). In contrast, no significant differences were observed between treated and non‐treated patients with full‐thickness lesions (ICRS III–IV) in terms of stability (5.3% vs. 5.3%; *p* = 1.000) or progression (21.1% vs. 10.5%; *p* = 0.660) (Table 3c).

### Survival and clinical outcomes

**Figure 2:**
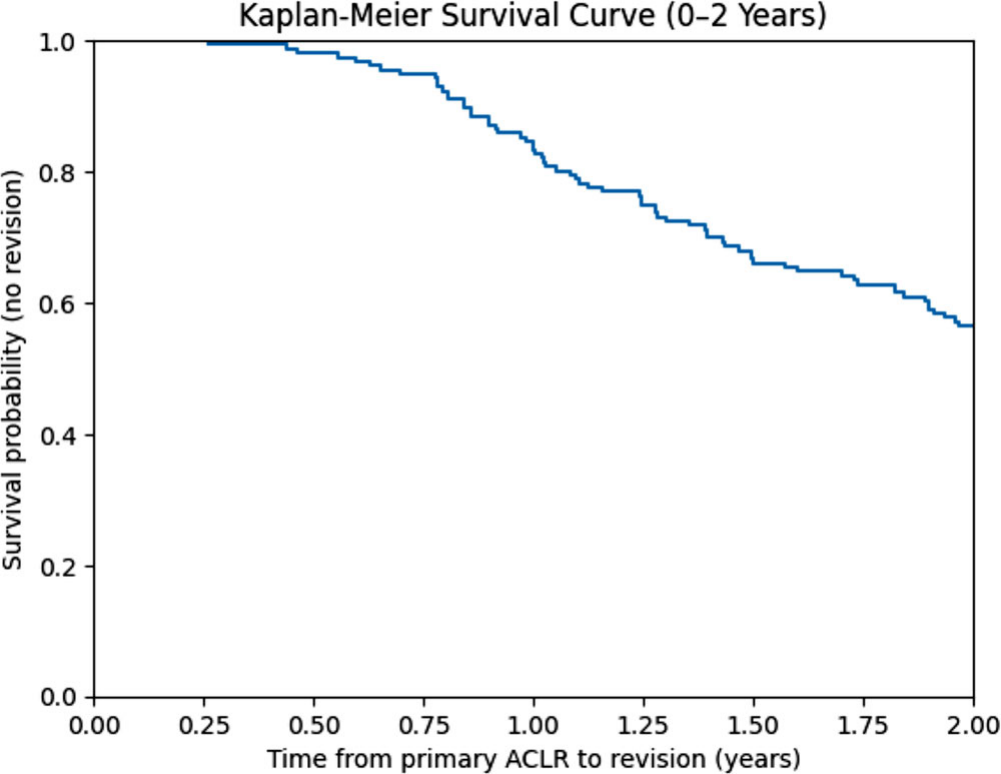
Kaplan–Meier survival curve illustrating revision‐free survival within the first 2 years after primary ACLR in the overall cohort. The estimated survival probability was 83% at 1 year and 56% at 2 years. ACLR, anterior cruciate ligament reconstruction.

A survival analysis was conducted to determine the rate of revision surgery within the first 2 years following primary ACLR. Overall, 26 of 156 patients (16.7%) underwent revision within the first year and 68 patients (43.6%) within 2 years after primary surgery, corresponding to an estimated survival probability of 83% at 1 year and 56% at 2 years (Figure 2).

When stratified according to cartilage treatment status, the proportion of early revisions (≤ 2 years) was comparable between groups. In the non‐treated group, 36 of 78 patients underwent revision within 2 years, compared with 32 of 78 patients in the treated group (log‐rank *p* = 0.53). Within the subgroup of patients who failed within 2 years (*n* = 68), lesion severity at primary surgery was predominantly low to intermediate grade. Fourteen patients had ICRS Grade I lesions, 37 Grade II, 11 Grade III and 6 Grade IV. The distribution of lesion grades within early failures did not differ between the two groups.

**Figure 3:**
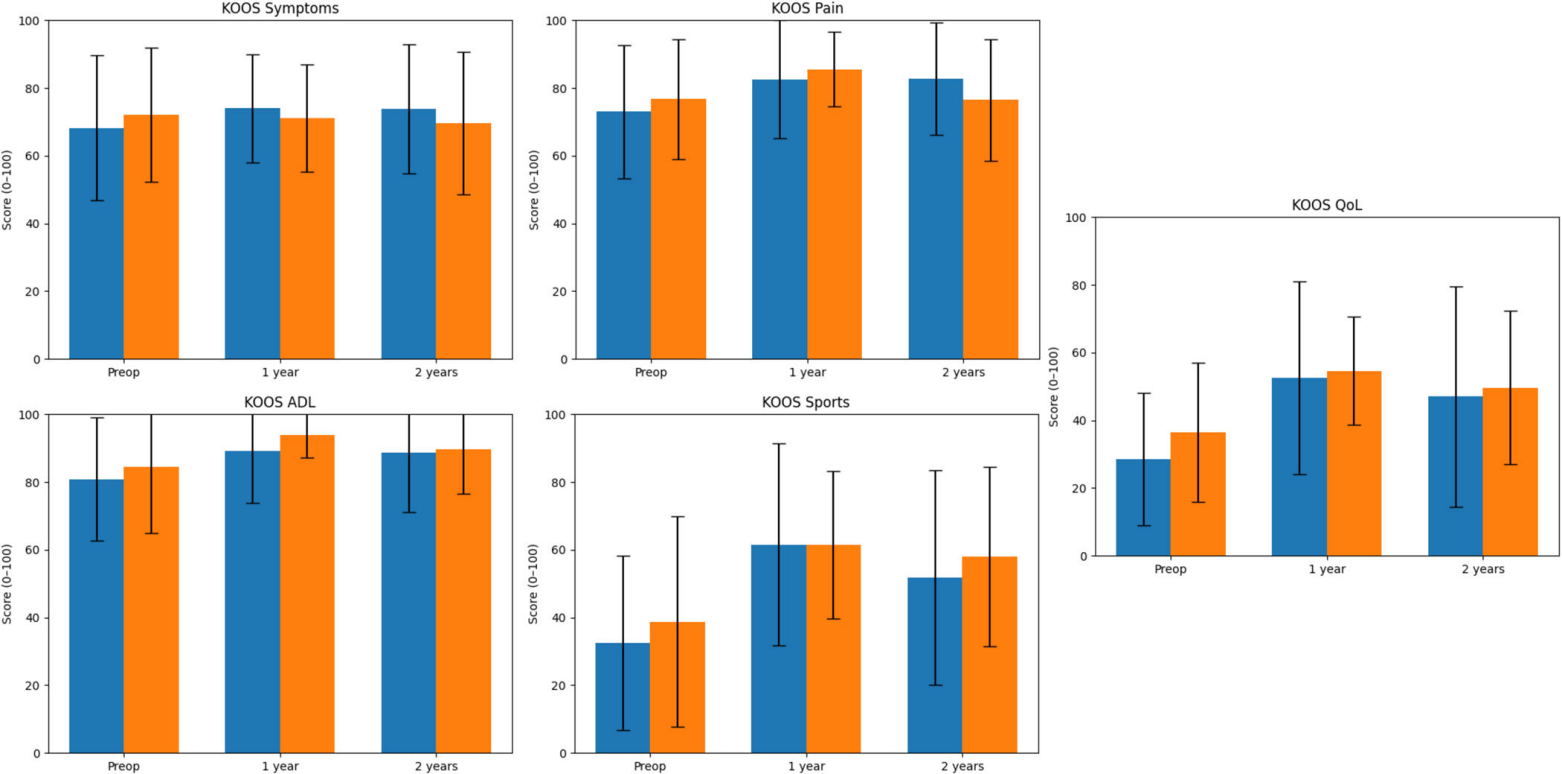
Evolution of KOOS subscales over time in patients with 2‐year follow‐up. Mean values (± standard deviation) of KOOS Symptoms, Pain, ADL, Sports and QoL are reported for the treated (blue bars) and non‐treated (orange bars) groups at preoperative, 1 year and 2 years. Both groups demonstrated significant improvement over time in most subscales, while no significant time × group interaction was observed, indicating comparable trajectories between groups. ADL, activities of daily living; KOOS, Knee Injury and Osteoarthritis Outcome Score; QoL, quality of life.

In contrast, 88 patients (56.4%) did not undergo revision within the first 2 years: this cohort included 46 treated patients and 42 non‐treated patients, with no significant difference in survival distribution between groups. These patients showed higher preoperative PROMs compared with those who underwent early revision ACLR, including KOOS Symptoms (70.0 ± 20.6 vs. 60.4 ± 20.8; *p* = 0.011), KOOS Pain (74.8 ± 18.7 vs. 66.8 ± 22.7; *p* = 0.042), KOOS activities of daily living (ADL) (82.5 ± 18.8 vs. 74.8 ± 22.9; *p* = 0.049), EQ‐5D (0.68 ± 0.22 vs. 0.58 ± 0.27; *p* = 0.050) and EQ‐VAS scores (59.7 ± 20.8 vs. 48.0 ± 27.2; *p* = 0.014), whereas no significant differences were observed for KOOS Sports or QoL. Moreover, baseline KOOS and EQ scores were comparable between treated and non‐treated patients. Significant improvements over time were observed in KOOS ADL, Sports and QoL in the treated group, and in KOOS Pain, ADL, Sports, QoL, EQ‐5D and EQ‐VAS in the non‐treated group. However, no significant time × group interaction was detected for any KOOS subscale, indicating similar clinical trajectories between treated and non‐treated patients over the 2‐year follow‐up. EQ‐5D and EQ‐VAS scores also improved over time in both groups, although a statistically significant interaction was observed for EQ‐5D (*p* = 0.046). KOOS scores are reported in Table 4 and illustrated in Figure 3.

**Table 4:** Clinical outcomes over time in treated and non‐treated groups with 2‐year follow‐up.

Outcome	Group	Preop	1 year	2 years	*p* Value (time effect, within group)	*p* Value (between groups)
KOOS Symptoms	Treated	68.2 ± 21.4	74.0 ± 16.0	73.9 ± 19.0	0.712	0.825
	Non‐treated	72.0 ± 19.8	71.1 ± 15.9	69.6 ± 21.1	0.457	
KOOS Pain	Treated	73.0 ± 19.6	82.6 ± 17.5	82.8 ± 16.6	0.057	0.545
	Non‐treated	76.7 ± 17.6	85.6 ± 10.9	76.5 ± 17.9	**0.003**	
KOOS ADL	Treated	80.8 ± 18.2	89.3 ± 15.4	88.7 ± 17.5	**0.033**	0.822
	Non‐treated	84.4 ± 19.5	94.0 ± 6.9	89.8 ± 13.2	**0.031**	
KOOS Sports	Treated	32.5 ± 25.8	61.5 ± 29.8	51.8 ± 31.8	**0.006**	0.678
	Non‐treated	38.8 ± 31.1	61.5 ± 21.8	58.1 ± 26.5	**0.005**	
KOOS QoL	Treated	28.5 ± 19.5	52.6 ± 28.5	47.0 ± 32.5	**0.014**	0.878
	Non‐treated	36.5 ± 20.5	54.6 ± 16.0	49.7 ± 22.6	**0.005**	
EQ‐5D	Treated	0.71 ± 0.19	0.78 ± 0.21	0.65 ± 0.31	0.086	**0.046**
	Non‐treated	0.64 ± 0.25	0.81 ± 0.12	0.79 ± 0.11	**0.016**	
EQ‐VAS	Treated	58.6 ± 21.5	72.8 ± 19.7	67.2 ± 23.8	0.056	0.314
	Non‐treated	60.9 ± 20.1	73.4 ± 20.8	72.6 ± 24.4	**0.038**	

*Note*: Data are expressed as mean ± standard deviation. *p* values refer to ANOVA for within‐group changes over time and between‐group comparisons. Values in bold indicate statistically significant differences (*p* < 0.05).

Abbreviations: ADL, activities of daily living; ANOVA, analysis of variance; EQ‐5D, EuroQol‐5 Dimensions; EQ‐VAS, EuroQol Visual Analogue Scale; KOOS, Knee Injury and Osteoarthritis Outcome Score; QoL, quality of life.

## DISCUSSION

The main finding of the present study is that surgical treatment of isolated cartilage lesions during primary ACLR was associated with a significantly higher rate of lesion stability at the time of revision ACLR compared to non‐treatment. However, no significant differences were observed in PROMs at 2‐year follow‐up.

This result stems from a matched‐pair study based on a nationwide registry, where the prevalence of cartilage lesions and predominant localization on the MFC aligned with prior epidemiological data in ACL‐injured populations [10, 19]. Despite their frequent occurrence during ACLR, the optimal management of these lesions, particularly when isolated and asymptomatic, remains controversial. While some evidence suggests that procedures such as microfracture may confer temporary structural benefit, others have reported inferior outcomes compared to non‐treatment, and concerns have been raised regarding iatrogenic damage to the subchondral bone [7, 18]. As a result, orthopaedic surgeons often face uncertainty in deciding whether and how to intervene on focal cartilage defects identified during ACLR. To address this gap, the present study assessed both structural progression and clinical impact of treating versus not treating isolated lesions during ACLR. Only focal defects were included, while patients with multi‐compartmental damage or major concomitant procedures were excluded to minimize confounding. The matching procedure resulted in well‐balanced cohorts with comparable lesion characteristics, including site, size and ICRS grade, thereby strengthening the internal validity of the comparison. A considerable proportion of lesions were low‐grade (ICRS I–II), which represent cartilage softening or partial‐thickness defects. Although low‐grade lesions are not always treated surgically in routine practice, minor procedures such as debridement may be performed at the surgeon's discretion when surface irregularities or unstable cartilage flaps are observed. For this reason, they are systematically recorded in the registry and reflect the full spectrum of cartilage findings encountered during ACLR [14, 30, 37].

A significantly higher rate of lesion stability was observed in the treated group compared to the non‐treated group (*p* < 0.05). Importantly, lesion regression was observed in more than 60% of cases in both groups, with no significant difference between treated and non‐treated patients. While overall progression rates were comparable between groups, a lower progression rate was observed in treated low‐grade lesions (*p* = 0.050). This may suggest a potential stabilizing effect of cartilage‐preserving procedures, a concept supported by both preclinical models and observational studies [11, 22, 35, 36]. Sugiu et al. [35] reported a notable increase in post‐traumatic cartilage damage at second‐look arthroscopy, indicating that untreated lesions may deteriorate even over relatively short follow‐up periods. Similarly, Hiranaka et al. [11] observed widespread chondral degeneration across multiple compartments at a mean of 15 months, despite clinically acceptable outcomes. In contrast, Gong et al. [8] and Nakamura et al. [21] described more heterogeneous trajectories, with persistent or worsening lesions in some cases, but also evidence of spontaneous healing in specific locations, particularly on the femoral condyles. These findings align with the structural deterioration observed in the untreated group of the present study and underscore concerns regarding the natural history of unaddressed cartilage damage. If left untreated, focal chondral defects may enlarge under ongoing mechanical load and inflammatory stress, eventually compromising adjacent cartilage and subchondral bone integrity [5, 12, 39]. This is particularly relevant in young and active individuals, where untreated cartilage lesions after ACL rupture have been associated with an increased risk of early PTOA and progressive radiographic degenerative changes over time, potentially affecting long‐term joint function and RTS capacity [4, 23, 24, 26]. Therefore, even in the absence of early symptomatic differences, structural progression may still carry prognostic implications and may justify selective treatment in specific patient subgroups.

Despite the greater lesion stability observed in the treated group at revision surgery, survival analysis showed no difference in revision rates between groups at 2‐year follow‐up (log‐rank *p* = 0.53). Moreover, PROMs were comparable between treated and non‐treated patients who had not experienced failure within 2 years. Similar findings were reported by Ulstein et al. [37] and Røtterud et al. [30], who observed no improvement in KOOS scores at 2–5 years with either procedure. More recently, Kjennvold et al. confirmed these results at 10‐year follow‐up in a binational registry cohort, showing no long‐term association between debridement or microfracture and better clinical outcomes [16]. These observations are further supported by a recent systematic review and meta‐analysis by Jevremovic et al., which reported no consistent clinical benefit of debridement or microfracture compared to non‐treatment [15]. The dissociation between structural findings and clinical outcomes observed in the present study further supports the concept that early cartilage changes may not immediately translate into patient‐perceived symptoms, particularly within a relatively short follow‐up period. Several hypotheses may explain these findings. A ‘ceiling effect’ may exist in young, athletic populations undergoing ACLR, where improvements in joint stability and neuromuscular function may dominate early postoperative recovery, potentially masking the effect of cartilage treatment on PROMs [38]. Moreover, the well‐established dissociation between structural cartilage damage and clinical symptoms, especially in early degenerative stages, may contribute to the lack of observable benefit [22]. Altogether, these findings challenge the utility of routine cartilage intervention in all cases of ACLR with concomitant cartilage lesions. Instead, they support a more selective approach, where surgical treatment may be reserved for specific lesion characteristics or patient subgroups.

This study presents several limitations that should be acknowledged. Firstly, the registry‐based design inherently limits causal inference and may be subject to variability in reporting surgical documentation. For instance, cartilage lesion size was available only as a categorical variable (<2 or ≥2 cm^2^), and exact lesion area measurements were not available. Secondly, treatment allocation was not randomized but based on the intraoperative discretion of the surgeon, introducing a potential selection bias, particularly related to lesion severity, size and location. Thirdly, the structural analysis inherently included only patients who subsequently underwent revision ACLR and therefore represents a selected population with graft failure rather than the overall ACLR population. Consequently, the structural findings should be interpreted as describing cartilage lesion evolution within a revision setting and may not be directly generalizable to stable ACLR knees. Moreover, while second‐look arthroscopy offers direct intra‐articular assessment and remains the clinical reference standard for cartilage surface evaluation [33], the lack of standardized imaging prevented correlation between surgical findings and radiological cartilage evolution. Intraoperative grading also remains vulnerable to interobserver variability. In addition, PROMs were limited to a 2‐year follow‐up, which may be insufficient to adequately assess the clinical impact of cartilage lesion progression or the potential delayed effects of cartilage treatment, and may also reflect symptoms related to residual instability, graft dysfunction or concomitant cartilage pathology. The registry does not provide standardized information regarding the mechanism of graft failure, including traumatic re‐injury, graft insufficiency or objective postoperative laxity measurements. Therefore, it was not possible to fully disentangle the relative contribution of instability, failure mechanism and cartilage status to lesion progression and PROMs. However, the matched cohorts demonstrated similar distributions of contact versus non‐contact sports participation, as well as comparable revision‐free survival and time to revision ACLR, indirectly suggesting a relatively balanced exposure to reinjury risk between groups. To partially mitigate this limitation, PROM analyses were restricted to patients who had not undergone revision within the first 2 years. Furthermore, data on RTS, which is an important outcome in the ACLR population, were not available. However, considering the general population included, the study was primarily designed to assess short‐term symptomatology and structural progression rather than high‐performance recovery metrics.

Despite these limitations, the study leveraged a large national registry and a rigorous matched‐pair design to minimize confounding and enable balanced comparisons. To our knowledge, this is the largest registry‐based investigation to directly compare treated versus untreated isolated cartilage lesions during ACLR, focusing on intra‐articular changes over time. By integrating second‐look arthroscopic data, it provides controlled comparative evidence of lesion progression and highlights a potential stabilizing effect of cartilage‐preserving procedures, offering novel insights into their biological and clinical implications. Further studies with longer follow‐up and imaging‐based assessments are needed to determine whether any structural benefits may translate into delayed osteoarthritic progression or improved long‐term outcomes.

## CONCLUSIONS

In this matched registry‐based study, surgical treatment of isolated cartilage lesions during primary ACLR was associated with higher structural lesion stability at revision surgery. However, this structural effect was not reflected in superior 2‐year PROMs among non‐revised patients. While these findings indicate a potential structural benefit, their clinical relevance remains uncertain and routine treatment of all isolated lesions during ACLR cannot be supported based on short‐term clinical results alone.

## AUTHOR CONTRIBUTIONS

*Conceptualization:* Iacopo Romandini and Alexander Sandon. *Methodology:* Iacopo Romandini and Piero Agostinone. *Formal analysis and investigation:* Iacopo Romandini and Alexander Sandon. *Writing—original draft preparation:* Iacopo Romandini. *Writing—review and editing:* Piero Agostinone, Pierre Rotzius and Alexander Sandon. *Supervision:* Alexander Sandon. All authors read and approved the final manuscript.

## CONFLICT OF INTEREST STATEMENT

The authors declare no conflicts of interest.

## ETHICS STATEMENT

The analysis of the Swedish ACL register is approved by the Regional Ethics Committee in Stockholm (ID 2011/337‐31/3). The registry complies with Swedish data security legislation. Participation in the Swedish Knee Ligament Registry is voluntary for both patients and surgeons, and written consent is not required for the use of anonymized national registry data in Sweden. The study was also approved by the SNKLR steering committee.

## Data Availability

The data that support the findings of this study are available from the corresponding author upon reasonable request.
